# Doppler Differential Positioning Technology Using the BDS/GPS Indoor Array Pseudolite System

**DOI:** 10.3390/s19204580

**Published:** 2019-10-21

**Authors:** Xingli Gan, Baoguo Yu, Lu Huang, Ruicai Jia, Heng Zhang, Chuanzhen Sheng, Guangwei Fan, Boyuan Wang

**Affiliations:** 1 State Key Laboratory of Satellite Navigation System and Equipment Technology, Shijiazhuang 050081, China; ganxingli@163.com (X.G.); yubg@sina.cn (B.Y.); jiaruicai@126.com (R.J.); 13582161539@163.com (H.Z.); shengchuanzhen@163.com (C.S.); fgweihb@163.com (G.F.); boyuan@hrbeu.edu.cn (B.W.); 2 The 54th Research Institute of China Electronics Technology Group Corporation, Shijiazhuang 050081, China

**Keywords:** Beidou satellite navigation system (BDS), indoor positioning, array pseudolite, Doppler differential positioning, dilution of precision, cycle-slip

## Abstract

A Global Satellite Navigation System (GNSS) cannot provide normal location services in an indoor environment because the signals are blocked by buildings. The Beidou satellite navigation system (BDS)/GPS indoor array pseudolite system is proposed to overcome the problems of indoor positioning with conventional pseudolite, such as time synchronization, ambiguity resolution and base stations. At the same time, an algorithm for Doppler differential positioning is proposed to improve the indoor positioning accuracy and the positioning coverage of the system, which uses the Doppler difference equation and Known Point Initialization (KPI) to determinate the velocity and position of the receiver. Experiments were conducted to verify the proposed system under different conditions; the average positioning error of the Doppler differential positioning algorithm was 7.86 mm in the kinematic test and 2.9 mm in the static test. The results show that BDS/GPS indoor array pseudolite system has the potential to make indoor positioning achieve sub-centimeter precision. Finally, the positioning error of the proposed algorithm is also analyzed, and the data tests show that the dilution of precision (DOP) and cycle- slips have a significant impact on the indoor positioning accuracy; a cycle-slip of a half-wavelength can cause positioning errors of tens of millimeters. Therefore, the Doppler-aided cycle-slip detection method (DACS) is proposed to detect cycle-slips of one cycle or greater than one, and the carrier phase double difference cycle-slip detection method (CPDD) is used to detect cycle slips of a half-wavelength.

## 1. Introduction

The Global Satellite Navigation System (GNSS) plays a leading role in outdoor positioning, but GNSS is unable to provide a normal location service in an indoor environment because the signal can be blocked by buildings. With the development of the Beidou satellite navigation system (BDS), Chinese researchers are developing a BDS/GPS indoor positioning pseudolite system to achieve seamless indoor and outdoor positioning services [1,2], the signals of which are received by commercialized GNSS chips such as unicorecomm UC6226 and ublox M8T. In addition, some pseudolites for indoor positioning have also been developed. The indoor messaging system (IMES) has been developed by the Japanese Aerospace Exploration Agency [3,4,5]; its positioning method is similar to Bluetooth, and its positioning accuracy usually ranges from 5 to 10 m. Two multi-channel pseudolite positioning systems have been proposed: one consists of three antennas which are located at the interval of the half-wavelength of the GPS L1 carrier wave—i.e., at 95.15 mm from each other—and is used to overcome the problems of time synchronization and indoor multipaths, but it cannot be used for dynamic positioning and has only a 4 m × 4 m coverage area [6]; the other is a combined approach of Doppler and carrier-based hyperbolic positioning with a multi-channel GPS pseudolite [7,8], in which the distance between transmitting antennas is not constrained by a half-wavelength, a Doppler observation equation is used to estimate the offset of the three-dimensional position and orientation, and a nonlinear observation equation for carrier phase difference is used to estimate ambiguity. Therefore, the disadvantage of the pseudolite is the complex ambiguity resolution and unstable indoor positioning results. A multi-channel pseudolite—which is called a GNSS repeater—has been proposed [9,10], which realizes indoor positioning using the carrier phase difference method with the base and rover stations. Some indoor positioning tests in Australia have also been carried out with the Locata pseudolite [11,12], which has the potential to allow point positioning with sub-centimeter precision (using carrier phase) for a mobile unit. Similarly, it also faces the problem of ambiguity resolution (AR).

Indoor environments are characterized by many reflectors in the path from the transmitter to the receiver, and the multipath error can be up to several tens of meters or even hundreds of meters [13,14,15]; some traditional ambiguity resolution methods, such as the ambiguity function method and least squares ambiguity decorrelation adjustment (LAMBDA) [16], usually fail to obtain fixed ambiguity. Therefore, the Known Point Initialization (KPI) method [17,18] has been adopted to solve the carrier phase ambiguity, which usually requires a higher precision of the known coordinates for the LAMBDA method. Due to the influence of bad DOP in indoor environments, this combination method of KPI and LAMBDA cannot pass the ambiguity validation, where the disadvantages of the complex computational process and discontinuous positioning results are not suitable for indoor pedestrian positioning based on smartphones.

In the following sections, the composition of the BDS/GPS indoor array pseudolite system is introduced, and a multi-channel pseudolite transmitter with the same clock source is used to reduce the complexity of time synchronization. In addition, an indoor positioning algorithm called Doppler differential positioning is proposed. Compared with Doppler positioning methods, the algorithm can reduce horizontal dilution of precision (HDOP) and increase positioning coverage. Furthermore, some static and kinematic tests are conducted to evaluate the positioning performance of the Doppler differential positioning algorithm. Finally, the positioning errors of the BDS/GPS indoor array pseudolite system are analyzed, and the Doppler-aided cycle-slip detection method (DACS) and the carrier phase double difference cycle-slip detection method (CPDD) are used to detect cycle-slips.

## 2. BDS/GPS Indoor Array Pseudolite System

**Figure 1:**
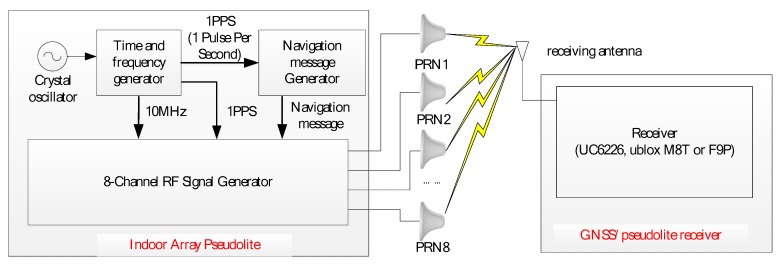
Composition of Beidou satellite navigation system (BDS)/GPS indoor array pseudolite system.

Figure 1 illustrates the composition of the BDS/GPS indoor array pseudolite system. It consists of three parts: An indoor array signal transmitter, an array antenna and a GNSS/pseudolite receiver. Because the coverage of the indoor pseudolite is much smaller than that of the outdoor pseudolite, a multi-channel transmitter with the same clock source is used to reduce the difficulty and complexity of time synchronization. Each channel transmits a signal with different spread spectrum codes, and the navigation message is modulated at 1575.42 MHz and 1561.098 MHz. The GNSS/pseudolite receiver mainly uses some commercial GNSS chips or smartphones, which can output the carrier phase and Doppler observations of the pseudolite [19]. Then, the position and speed of the receiver can be calculated by the indoor positioning software using the Doppler differential method.

**Figure 2:**
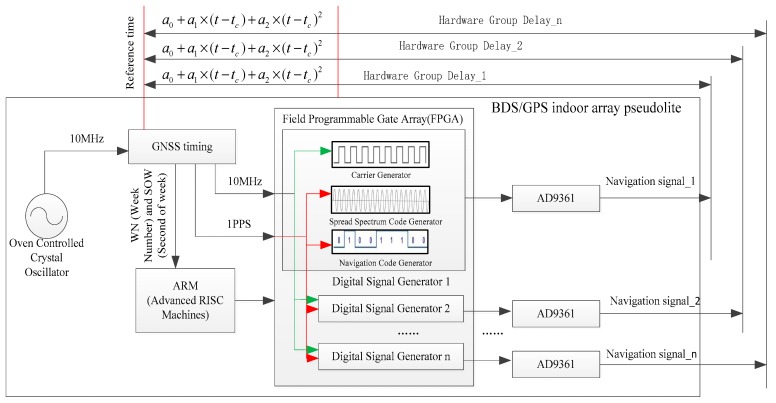
Time synchronization method of BDS/GPS indoor array pseudolite. GNSS: Global Satellite Navigation System.

Figure 2 illustrates the time synchronization method of the BDS/GPS indoor array pseudolite. The pseudolite time is in reference to GPS time, although the pseudolite clock and GPS time are different. Since signals of the pseudolite array are generated at the same 1 Pulse Per Second (1PPS), the pseudolite clock biases are as follows:

(1) $${\mathrm{dt}}^{s}=a_{0}+a_{1}\times (t-t_{c})+a_{2}\times {\left(t-t_{c}\right)}^{2}+\tau$$

where ${\mathrm{dt}}^{s}$ is the pseudolite clock biases, $a_{0}$ is the time offset of satellite clock errors, $a_{1}$ is the satellite clock rate coefficient of deviation, $a_{2}$ is the drift coefficient of the satellite clock speed rate, τ is the hardware group delay, $t_{c}$ is the reference moment, and t is the calculation moment of the pseudolite clock error.

For a multi-channel pseudolite system, the clock biases of each channel can be expressed by

(2) $$\{\begin{array}{c} {\mathrm{dt}}_{1}^{s}=a_{0}+a_{1}\times (t-t_{c})+a_{2}\times {\left(t-t_{c}\right)}^{2}+\tau_{1}\\ {\mathrm{dt}}_{2}^{s}=a_{0}+a_{1}\times (t-t_{c})+a_{2}\times {\left(t-t_{c}\right)}^{2}+\tau_{2}\\ \ldots \ldots \\ {\mathrm{dt}}_{n}^{s}=a_{0}+a_{1}\times (t-t_{c})+a_{2}\times {\left(t-t_{c}\right)}^{2}+\tau_{n} \end{array}$$

where n is number of channels, and $\tau_{1}$, $\tau_{2}$, ……, $\tau_{n}$ are the hardware group delays of multi-channel pseudolite.

The rate of pseudolite clock biases $d{\dot{t}}^{s}$ is as follows:

(3) $$\{\begin{array}{c} d{\dot{t}}_{1}^{s}=a_{1}+a_{2}\times (t-t_{c})\\ d{\dot{t}}_{1}^{s}=a_{1}+a_{2}\times (t-t_{c})\\ \ldots \ldots \\ d{\dot{t}}_{1}^{s}=a_{1}+a_{2}\times (t-t_{c}) \end{array}$$

Therefore, Doppler observations are used to calculate the velocity and position of the receiver, which only uses the rate of pseudolite clock biases; then, it can be observed that there is no time synchronization error in the BDS/GPS indoor array pseudolite.

## 3. Methodology

The mathematical basics of the Doppler observation equation [20], Doppler differential observation equation, position determination and dilution of precision are presented in detail in this section.

### 3.1. Doppler Observation and Positioning Equation

The Doppler observation equation between the receiver *u* and the channel *i* of the indoor array pseudolite can be expressed by

(4) $$\frac{c}{f}\cdot D_{u}^{i}={\dot{R}}_{u}^{i}+c\cdot d{\dot{t}}_{u}-c\cdot d{\dot{t}}^{s}+{\dot{\varepsilon }}_{u}^{i}$$

where $D_{u}^{i}$ is the Doppler measurement between the receiver *u* and transmitting channel of the indoor array pseudolite; *c* is the speed of light; *f* is the frequency of navigation signal; ${\dot{R}}_{u}^{i}$ is the change rate of geometric distance between receiver u and transmitting antenna i of the pseudolite; $d{\dot{t}}_{u}$ is the rate of receiver clock biases; $d{\dot{t}}^{s}$ is the rate of pseudolite clock biases; and ${\dot{\varepsilon }}_{u}^{i}$ is the combined error residual, which mainly includes the antenna phase center deviation, multipath error and thermal noise.

The rate of geometric distance can be calculated by

(5) $$\begin{array}{l} {\dot{R}}_{u}^{i}=\frac{(x_{u}-x^{i})\times {\dot{x}}_{u}+(y_{u}-y^{i})\times {\dot{y}}_{u}+(z_{u}-z^{i})\times {\dot{z}}_{u}}{\sqrt{{\left(x_{u}-x^{i}\right)}^{2}+{\left(y_{u}-y^{i}\right)}^{2}+{\left(z_{u}-z^{i}\right)}^{2}}}\\ \;\;\;\;\;\;=\left[\begin{array}{ccc} e_{x}^{i} & e_{y}^{i} & e_{z}^{i} \end{array}\right]\times \left[\begin{array}{c} {\dot{x}}_{u}\\ {\dot{y}}_{u}\\ {\dot{z}}_{u} \end{array}\right] \end{array}$$

where $x_{u}$, $y_{u}$ and $z_{u}$ are the three-dimensional coordinates of the receiver; ${\dot{x}}_{u}$, ${\dot{y}}_{u}$ and ${\dot{z}}_{u}$ are the velocity of the receiver, which can be solved with four Doppler observations and least squares estimation; and $x^{i}$, $y^{i}$ and $z^{i}$ are the three-dimensional positions of the pseudolite transmitting channel. $\left[\begin{array}{ccc} e_{x}^{i} & e_{y}^{i} & e_{z}^{i} \end{array}\right]$ is called a geometry matrix.

The Doppler observation equations of the indoor array pseudolite can be expressed as the following matrix form:

(6) $$\left[\begin{array}{cccc} e_{x}^{1} & e_{y}^{1} & e_{z}^{1} & 1\\ e_{x}^{2} & e_{y}^{2} & e_{z}^{2} & 1\\ \ldots & \ldots & \ldots & \ldots \\ e_{x}^{n} & e_{y}^{n} & e_{z}^{n} & 1 \end{array}\right]\cdot \left[\begin{array}{c} {\dot{x}}_{u}\\ {\dot{y}}_{u}\\ {\dot{z}}_{u}\\ c\cdot d{\dot{t}}_{u}-c\cdot d{\dot{t}}^{s} \end{array}\right]=\left[\begin{array}{c} \frac{c}{f}\cdot D_{u}^{1}\\ \frac{c}{f}\cdot D_{u}^{2}\\ \ldots \\ \frac{c}{f}\cdot D_{u}^{n} \end{array}\right]+\left[\begin{array}{c} {\dot{\varepsilon }}_{u}^{1}\\ {\dot{\varepsilon }}_{u}^{2}\\ \ldots \\ {\dot{\varepsilon }}_{u}^{n} \end{array}\right]$$

### 3.2. Doppler Differential Observation and Positioning Equation

The Doppler observation equation of pseudolite channels *i* and *j* can be denoted by

(7) $$\{\begin{array}{c} \frac{c}{f}\cdot D_{u}^{i}={\dot{R}}_{u}^{i}+c\cdot d{\dot{t}}_{u}-c\cdot d{\dot{t}}^{s}+{\dot{\varepsilon }}_{u}^{i}\\ \frac{c}{f}\cdot D_{u}^{j}={\dot{R}}_{u}^{j}+c\cdot d{\dot{t}}_{u}-c\cdot d{\dot{t}}^{s}+{\dot{\varepsilon }}_{u}^{j} \end{array}$$

The difference equation of the Doppler observations between channel *i* and *j* can eliminate the rate of receiver and pseudolite clock biases, which can be written as follows:

(8) $$\frac{c}{f}\cdot \Delta D_{u}^{i,j}=\Delta {\dot{R}}_{u}^{i,j}+\Delta {\dot{\varepsilon }}_{u}^{i,j}$$

where $\Delta D_{u}^{i,j}$ represents the difference of the Doppler measurement, $\Delta {\dot{R}}_{u}^{i,j}$ represents the difference in the rate of geometric distance, and $\Delta {\dot{\varepsilon }}_{u}^{i,j}$ represents the error of the Doppler differential observation. The differential Doppler positioning equation of the indoor array pseudolite is denoted by

(9) $$\left[\begin{array}{ccc} e_{x}^{2}-e_{x}^{1} & e_{y}^{2}-e_{y}^{1} & e_{z}^{2}-e_{z}^{1}\\ e_{x}^{3}-e_{x}^{1} & e_{y}^{3}-e_{y}^{1} & e_{z}^{3}-e_{z}^{1}\\ \ldots & \ldots & \ldots \\ e_{x}^{n}-e_{x}^{1} & e_{y}^{n}-e_{y}^{1} & e_{z}^{n}-e_{z}^{1} \end{array}\right]\times \left[\begin{array}{c} {\dot{x}}_{u}\\ {\dot{y}}_{u}\\ {\dot{z}}_{u} \end{array}\right]=\left[\begin{array}{c} \frac{c}{f}\cdot \Delta D_{u}^{2,1}\\ \frac{c}{f}\cdot \Delta D_{u}^{3,1}\\ \ldots \\ \frac{c}{f}\cdot \Delta D_{u}^{n,1} \end{array}\right]+\left[\begin{array}{c} \Delta {\dot{\varepsilon }}_{u}^{2,1}\\ \Delta {\dot{\varepsilon }}_{u}^{3,1}\\ \ldots \\ \Delta {\dot{\varepsilon }}_{u}^{n,1} \end{array}\right]$$

where *n* is the number of transmission channels and is greater than or equal to 4.

### 3.3. Position Determination

The matrix on the left-hand side of Equations (5) and (8) is defined as A, and the two column vectors on the right-hand side are, respectively, defined as b (left one) and ε (right one); then, Equations (6) or (9) can be written as

(10) $$A\cdot v_{u,s}=b+\varepsilon$$

If an initial value of $v_{u}$ is used for the solution-updating process, the Newton–Raphson method is described as $v_{u,0}=({\dot{x}}_{u,0},{\dot{y}}_{u,0},{\dot{z}}_{u,0})$. The least squares updated solution can be represented as

(11) $$\Delta v_{u,0}={\left(A^{T}A\right)}^{-1}A^{T}b$$

Then, the velocity can be updated iteratively according to

(12) $${\hat{v}}_{u,1}=v_{u,0}+\Delta v_{u,0}$$

The position $r_{u,1}$ is denoted by

(13) $$r_{u,1}=r_{u,0}+{\hat{v}}_{u,1}\cdot \Delta t$$

where $r_{u,0}$ is the initial value of $r_{u}$, which is the coordinate of receiver *u*, and Δt represents the time interval of the observation.

Here, the Doppler differential positioning algorithm is summarized in Algorithm 1. In Known Point Initialization (KPI), the starting point of the receiver is initialized, whose three-dimensional (3D) coordinates are measured by a total station. The least squares method is used to determinate the velocity of the user receiver. The receiver position for the current epoch is calculated according to Equation (13). The starting point of the receiver is assigned to the position of the current epoch, whose position and velocity are calculated again.


**Algorithm 1**
1Initialize the known point of GNSS/pseudolite receiver and z is a constant2continue = true3**while** continue **do**4  velocity at epoch t = velocity at epoch t+15  position at epoch t = position at epoch t+16  **for** number of available channels **do**7    Calculate e_x_ and e_y_ for each available channel8  **end**9  **for** number of available channels - 1 **do**10    Calculate the matrix **A**11    Calculate the Doppler differential matrix ***b***12  **end**13  $\Delta v={\left(A^{T}A\right)}^{-1}A^{T}b$.14  velocity at epoch t+1 = velocity at epoch t +Δv.15  Calculate the position at epoch t+116**If**Δv≤0.0001 **then**17   break;18
**end**
19
**end**


It is difficult to get a good geometric distribution for the BDS/GPS indoor array pseudolite; the worst case is that all transmitting antennas are on a horizontal plane, HDOP is particularly high, and the least squares updated solution according to Equation (11) cannot converge. Therefore, the z-coordinate is set as a constant for pedestrian navigation.

The initialization (KPI) can be obtained by Quick Response (QR) codes on the ground, using visual location in actual system, whose three-dimensional (3D) coordinates are measured in advance by a total station. They are stored in the database together with the coordinates of the transmitting antenna.

### 3.4. Dilution of Precision

The dilution of precision (DOP) [21,22] can be expressed as

(14) $$\mathrm{cov}(\Delta v_{u})=\sigma_{\Delta \dot{\varepsilon }}^{2}\cdot {\left(A^{T}A\right)}^{-1}$$

If ${\left(A^{T}A\right)}^{-1}$ is defined as H, the HDOP can be expressed as the diagonal elements of H.

The diagonal elements of H of the Doppler positioning method are as follows:

(15) $$H=\left[\begin{array}{cccc} v_{x}DOP^{2} & & & \\ & v_{y}DOP^{2} & & \\ & & v_{z}DOP^{2} & \\ & & & v_{t}DOP^{2} \end{array}\right]$$

The diagonal elements of H of the Doppler differential positioning method are as follows:

(16) $$H=\left[\begin{array}{ccc} v_{x}DOP^{2} & & \\ & v_{y}DOP^{2} & \\ & & v_{z}DOP^{2} \end{array}\right]$$

The DOP for the x-y plane is defined as HDOP,

(17) $$HDOP=\sqrt{v_{x}DOP^{2}+v_{y}DOP^{2}}$$

where $v_{x}DOP^{2}$ means the DOP for the x-coordinate and likewise for the y- and z-coordinates.

## 4. Implementations and Evaluation

**Figure 3:**
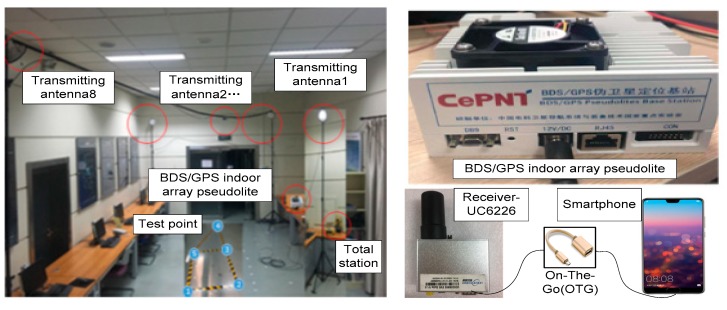
Experimental environments and setup.

In the BDS/GPS indoor array pseudolite system, static and kinematic tests were conducted to evaluate the performance of indoor positioning using the Doppler differential positioning algorithm. An indoor positioning testbed was built in a room as shown in Figure 3. The BDS/GPS indoor array pseudolite has eight output signals, which are connected to eight transmitting antennas by cables, and eight transmitting antennas’ coordinates are measured in advance by a total station, as shown in Table 1. The UBLOX NEO-M8T is used to receive the pseudolite’s signals and can provide access to raw measurements. Finally, the position and speed of the receiver are computed on a smartphone. The size of the test area is about 9 m long and 7 m wide. Five test points are selected on the ground, and their coordinates are measured by a total station. Point 3 is selected for the static positioning accuracy test. To compare the performance between the Doppler positioning algorithm and Doppler differential positioning algorithm in a kinematic test situation, a straight line (test path A) composed of point 2 and point 4 and a rectangle (Test path B) composed of point 1, point 2, point 3 and point 5 are used. 

### 4.1. Static Test Results

In the static test situations, the first situation is used analyze the performance of the Doppler differential measurement, and the other is used to test the positioning accuracy of the Doppler differential positioning algorithm for the BDS/GPS indoor array pseudolite. If the velocity of the receiver is zero, Equation (9) can be written as

(18) $$\left[\begin{array}{c} \frac{c}{f}\cdot \Delta D_{u}^{2,1}\\ \frac{c}{f}\cdot \Delta D_{u}^{3,1}\\ \ldots \\ \frac{c}{f}\cdot \Delta D_{u}^{n,1} \end{array}\right]+\left[\begin{array}{c} \Delta {\dot{\varepsilon }}_{u}^{2,1}\\ \Delta {\dot{\varepsilon }}_{u}^{3,1}\\ \ldots \\ \Delta {\dot{\varepsilon }}_{u}^{n,1} \end{array}\right]=0\;\mathrm{and}\;\frac{c}{f}\cdot \Delta D_{u}^{n,1}=-\Delta {\dot{\varepsilon }}_{u}^{n,1}$$

where 0 is a zero vector, $0={\left[0,0,0,\ldots ,0\right]}^{T}$. If the Doppler differential measurement noise $\Delta {\dot{\varepsilon }}_{u}^{n,1}$ is zero, $\Delta D_{u}^{n,1}$ is zero. Therefore, the standard deviation (std) of $\Delta {\dot{\varepsilon }}_{u}^{n,1}$ can be written as Equation (19), which represents the performance of Doppler differential measurement.

(19) $$\sigma_{\Delta {\dot{\varepsilon }}_{n,1}}=\mathrm{std}(\Delta D_{u}^{n,1})$$

**Figure 4:**
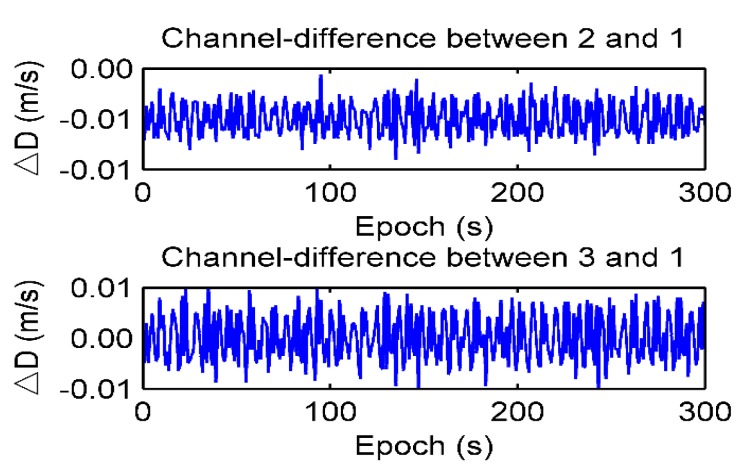
Doppler differential measurements in the static test.

The Doppler differential measurements in a static test are shown in Figure 4 and Table 2. The average value (AVG) of Doppler differential measurement is from 9 × 10^−6^ m/s to 4 × 10^−5^ m/s, and the standard deviation (STD) of Doppler differential measurement is from 0.0025 m/s to 0.005 m/s.

**Figure 5:**
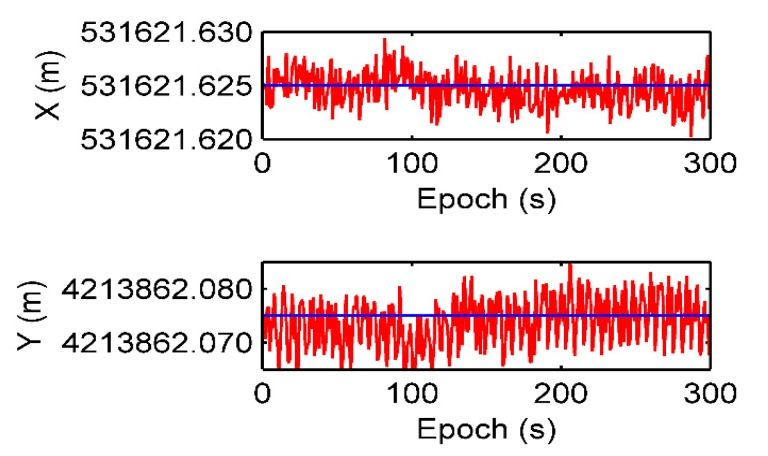
Positioning results using the Doppler differential positioning algorithm in the static test.

**Figure 6:**
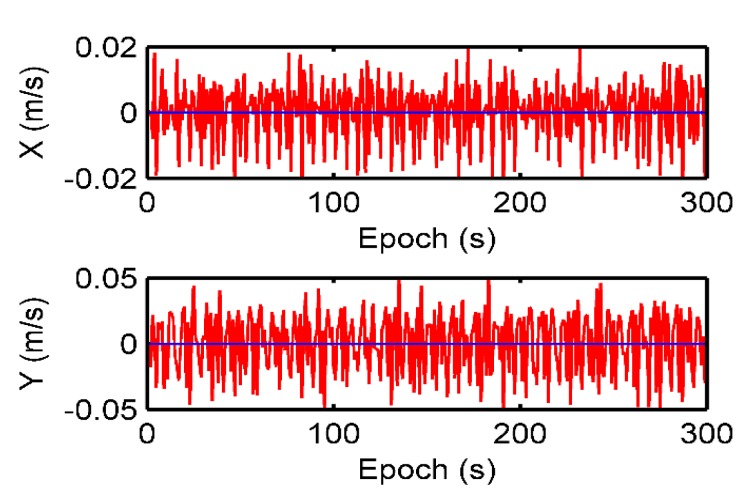
Velocity estimation results using the Doppler differential positioning algorithm in the static test.

The positioning and velocity results using the Doppler differential algorithm in the static test are shown in Figure 5 and Figure 6. The average positioning error is 0.0009 m in the X axis and 0.0028 m in the Y axis, respectively. It can be seen that the average velocity error is 0.06 × 10^−3^ m/s in X axis and 0.11 × 10^−3^ m/s in Y axis. The standard deviations of the positioning errors for the X-axis and Y-axis are 0.0034 m and 0.0097 m, respectively. The standard deviations of the velocity errors for the X-axis and Y-axis are 0.0098 m/s and 0.0245 m/s, respectively. It can be concluded that the accuracy of the Doppler differential measurement obtained by the static positioning test is at the millimeter level, and its positioning is also at the sub-centimeter level. 

### 4.2. Kinematic Test Results

**Figure 7:**
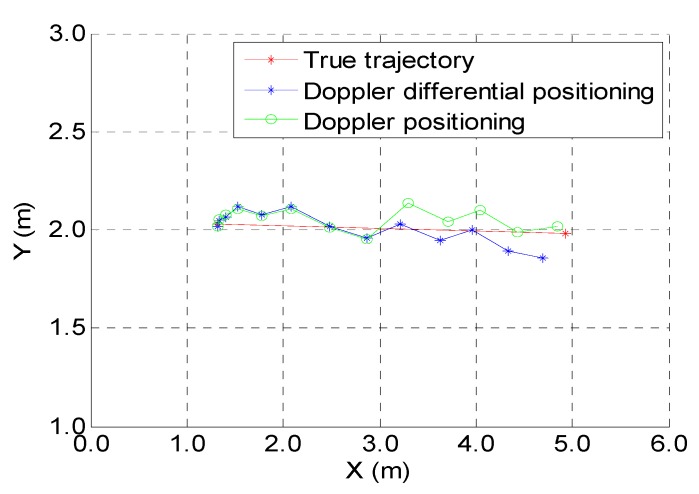
Kinematic test on a straight-line trajectory.

Figure 7 shows the kinematic test on a straight-line trajectory during human walking with a BDS/pseudolite receiver from point 2 to point 4; it can be found that the positioning accuracy of the two algorithms is the same in the first eight epochs. Table 3 shows the kinematic positioning errors; the average positioning error is 7.07 mm for the Doppler differential positioning algorithm, and 21.07 mm for the Doppler positioning algorithm.

**Figure 8:**
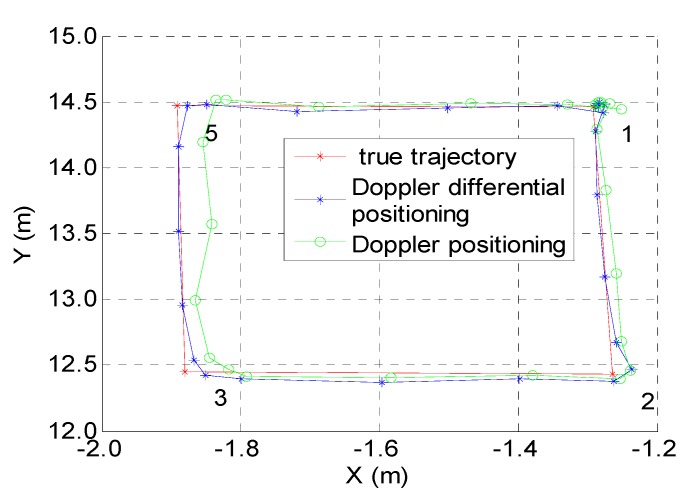
Kinematic test on a square trajectory.

Figure 8 shows the positioning results of the two algorithms mentioned above; the red line is the actual trajectory, the blue line is the estimated trajectory of the Doppler differential positioning algorithm, and the green line is the estimated trajectory of the Doppler positioning algorithm. Table 4 shows the kinematic positioning errors on a straight-line trajectory; the average positioning error is 20.83 mm for the Doppler differential positioning algorithm, and this value is 40.4 mm for the Doppler positioning algorithm.

## 5. Positioning Error Analysis

As indicated in Equation (14), the positioning errors of the two algorithms mentioned above are related to the measurement error and the dilution of precision. The accuracy of the Doppler measurement is at the mm/s level [23]. Meanwhile, the accuracy of the Doppler differential measurement obtained by the static positioning test is also at the millimeter level. Whether the Doppler differential positioning algorithm is better than the Doppler positioning algorithm in reducing positioning error, and whether the Doppler differential positioning algorithm can improve the horizontal dilution of precision, are further discussed below. The measurement error caused by cycle-slip will have a significant impact on positioning accuracy; thus, some cycle-slip detection methods are required.

### 5.1. Deviation of the Receiver Antenna Coordinate

**Figure 9:**
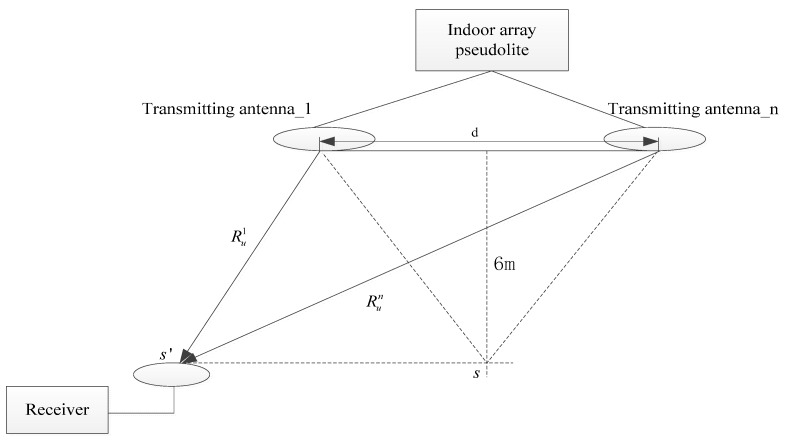
Geometric relationship between the transmitting antennas and receiver.

During the initialization of known points and the location processing, there will be some deviations in the antenna coordinates of the receiver, as shown in Figure 9. If the receiver moves from s to s’, then $R_{u}^{1}\leq R_{u}^{n}$, where $R_{u}^{1}$ is the distance from the receiver to transmitting antenna 1, and $R_{u}^{n}$ is the distance from the receiver to transmitting antenna *n*. Assuming that the error of the receiver antenna coordinate is $\left[\begin{array}{ccc} \Delta x_{u} & \Delta y_{u} & \Delta z_{u} \end{array}\right]$, and other measurement errors are ignored, Equation (5) can be expressed as the following matrix form:

(20) $$\begin{array}{l} R_{u}^{i}=\frac{(x_{u}+\Delta x_{u}-x^{i})\times {\dot{x}}_{u}+(y_{u}+\Delta y_{u}-y^{i})\times {\dot{y}}_{u}+(z_{u}+\Delta z_{u}-z^{i})\times {\dot{z}}_{u}}{\sqrt{{\left(x_{u}+\Delta x_{u}-x^{i}\right)}^{2}+{\left(y_{u}+\Delta y_{u}-y^{i}\right)}^{2}+{\left(z_{u}+\Delta z_{u}-z^{i}\right)}^{2}}}\;\\ =\left[\frac{(x_{u}-x^{i})\times {\dot{x}}_{u}+(y_{u}-y^{i})\times {\dot{y}}_{u}+(z_{u}-z^{i})\times {\dot{z}}_{u}}{R_{u}^{i}}+\frac{\Delta x_{u}\times {\dot{x}}_{u}+\Delta y_{u}\times {\dot{y}}_{u}+\Delta z_{u}\times {\dot{z}}_{u}}{R_{u}^{i}}\right]\\ =\left[\begin{array}{ccc} e_{x}^{i}+\frac{\Delta x_{u}}{R_{u}^{i}} & e_{y}^{i}+\frac{\Delta y_{u}}{R_{u}^{i}} & e_{z}^{i}+\frac{\Delta z_{u}}{R_{u}^{i}} \end{array}\right]\times \left[\begin{array}{c} {\dot{x}}_{u}\\ {\dot{y}}_{u}\\ {\dot{z}}_{u} \end{array}\right]=\left[\begin{array}{ccc} e_{x}^{i} & e_{y}^{i} & e_{z}^{i} \end{array}\right]\times \left[\begin{array}{c} {\dot{x}}_{u}\\ {\dot{y}}_{u}\\ {\dot{z}}_{u} \end{array}\right]+\left[\begin{array}{ccc} \frac{\Delta x_{u}}{R_{u}^{i}} & \frac{\Delta y_{u}}{R_{u}^{i}} & \frac{\Delta z_{u}}{R_{u}^{i}} \end{array}\right]\times \left[\begin{array}{c} {\dot{x}}_{u}\\ {\dot{y}}_{u}\\ {\dot{z}}_{u} \end{array}\right] \end{array}$$

The Doppler observation equations (Equation (6)) can be written as

(21) $$\left[\begin{array}{ccc} e_{x}^{1} & e_{y}^{1} & e_{z}^{1}\\ e_{x}^{2} & e_{y}^{2} & e_{z}^{2}\\ \ldots & \ldots & \ldots \\ e_{x}^{n} & e_{y}^{n} & e_{z}^{n} \end{array}\right]\cdot \left[\begin{array}{c} {\dot{x}}_{u}\\ {\dot{y}}_{u}\\ {\dot{z}}_{u} \end{array}\right]+\underset{B}{\underset{︸}{\left[\begin{array}{ccc} \frac{\Delta x_{u}}{R_{u}^{1}} & \frac{\Delta y_{u}}{R_{u}^{1}} & \frac{\Delta z_{u}}{R_{u}^{1}}\\ \frac{\Delta x_{u}}{R_{u}^{2}} & \frac{\Delta y_{u}}{R_{u}^{2}} & \frac{\Delta z_{u}}{R_{u}^{2}}\\ \ldots & \ldots & \ldots \\ \frac{\Delta x_{u}}{R_{u}^{n}} & \frac{\Delta y_{u}}{R_{u}^{n}} & \frac{\Delta y_{u}}{R_{u}^{n}} \end{array}\right]\cdot \left[\begin{array}{c} {\dot{x}}_{u}\\ {\dot{y}}_{u}\\ {\dot{z}}_{u} \end{array}\right]}}=\left[\begin{array}{c} \frac{c}{f}\cdot D_{u}^{1}-(c\cdot d{\dot{t}}_{u}-c\cdot d{\dot{t}}^{s})\\ \frac{c}{f}\cdot D_{u}^{2}-(c\cdot d{\dot{t}}_{u}-c\cdot d{\dot{t}}^{s})\\ \ldots \\ \frac{c}{f}\cdot D_{u}^{n}-(c\cdot d{\dot{t}}_{u}-c\cdot d{\dot{t}}^{s}) \end{array}\right]$$

where B is the error residual for the Doppler observation equations, which is caused by the deviation of the receiver antenna coordinate.

The differential positioning equation (Equation (9)) is denoted by

(22) $$\left[\begin{array}{ccc} e_{x}^{2}-e_{x}^{1} & e_{y}^{2}-e_{y}^{1} & e_{z}^{2}-e_{z}^{1}\\ e_{x}^{3}-e_{x}^{1} & e_{y}^{3}-e_{y}^{1} & e_{z}^{3}-e_{z}^{1}\\ \ldots & \ldots & \ldots \\ e_{x}^{n}-e_{x}^{1} & e_{y}^{n}-e_{y}^{1} & e_{z}^{n}-e_{z}^{1} \end{array}\right]\times \left[\begin{array}{c} {\dot{x}}_{u}\\ {\dot{y}}_{u}\\ {\dot{z}}_{u} \end{array}\right]+\underset{C}{\underset{︸}{\left[\begin{array}{ccc} \frac{\Delta x_{u}\cdot (R_{u}^{2}-R_{u}^{1})}{R_{u}^{1}R_{u}^{2}} & \frac{\Delta y_{u}\cdot (R_{u}^{2}-R_{u}^{1})}{R_{u}^{1}R_{u}^{2}} & \frac{\Delta z_{u}\cdot (R_{u}^{2}-R_{u}^{1})}{R_{u}^{1}R_{u}^{2}}\\ \frac{\Delta x_{u}\cdot (R_{u}^{3}-R_{u}^{1})}{R_{u}^{1}R_{u}^{3}} & \frac{\Delta y_{u}\cdot (R_{u}^{3}-R_{u}^{1})}{R_{u}^{1}R_{u}^{3}} & \frac{\Delta z_{u}\cdot (R_{u}^{3}-R_{u}^{1})}{R_{u}^{1}R_{u}^{3}}\\ \ldots & \ldots & \ldots \\ \frac{\Delta x_{u}\cdot (R_{u}^{n}-R_{u}^{1})}{R_{u}^{1}R_{u}^{n}} & \frac{\Delta y_{u}\cdot (R_{u}^{n}-R_{u}^{1})}{R_{u}^{1}R_{u}^{n}} & \frac{\Delta z_{u}\cdot (R_{u}^{n}-R_{u}^{1})}{R_{u}^{1}R_{u}^{n}} \end{array}\right]\cdot \left[\begin{array}{c} {\dot{x}}_{u}\\ {\dot{y}}_{u}\\ {\dot{z}}_{u} \end{array}\right]}}=\left[\begin{array}{c} \frac{c}{f}\cdot \Delta D_{u}^{2,1}\\ \frac{c}{f}\cdot \Delta D_{u}^{3,1}\\ \ldots \\ \frac{c}{f}\cdot \Delta D_{u}^{n,1} \end{array}\right]$$

where C is the error residual for the differential positioning equation, which is caused by the deviation of the receiver antenna coordinate.

Although the residual equations B and C are treated nonlinearly by the least squares updated solution in Equation (11), it is very difficult to analyze the error by a mathematical method. However, some very useful conclusions can be reached: 1) if the receiver is stationary, then,$\Delta D_{u}^{2,1}\approx \Delta D_{u}^{3,1}\approx \ldots \ldots \approx \Delta D_{u}^{n,1}$, and the speed of the receiver is not affected by the deviation of the receiver antenna coordinate; and 2) the deviation $\left[\begin{array}{ccc} \Delta x_{u} & \Delta y_{u} & \Delta z_{u} \end{array}\right]$ needs to be much smaller than the distance from the receiver to the transmitting antenna, otherwise the least squares updated equation will not converge.

According to Table 1, if the distance between two transmitting antennas is less than or equal to 3 m, then $R_{u}^{n}-R_{u}^{1}\leq 3$, and because $R_{u}^{n}\geq R_{u}^{1}\geq 6m$ (the height between the transmitting antenna and the ground is 6 m), $(R_{u}^{n}-R_{u}^{1})/(R_{u}^{1}R_{u}^{n})\leq 3/(6\cdot R_{u}^{n})\leq 1/(2\cdot R_{u}^{n})\leq 1/R_{u}^{n}$, In order to prove the above analysis results, the x-error or y-error is manually added to the coordinates of KPI ($\Delta x_{u}$ = 4.0 m, $\Delta y_{u}$ = 4.0 m). The velocity of the receiver is calculated as ($\Delta x_{u}$ = 4.0 m, $\Delta y_{u}$ = 4.0 m) and ($\Delta x_{u}$ = 0 m, $\Delta y_{u}$ = 0 m) and the velocity measurement error can be written as

(23) $$\Delta {V=V}_{(\Delta x_{u}=4.0m,\Delta y_{u}=4.0m)}{-V}_{(\Delta x_{u}=0m,\Delta y_{u}=0m)}$$

The velocity measurement error is greatly increased in the 24th epoch of Table 5, and the least squares updated equation will not converge in the 25th epoch of the Doppler positioning method. Therefore, the differential positioning method is more tolerant to the deviation in the antenna coordinates of the receiver than the Doppler positioning method. Under the same deviation conditions, the velocity measurement accuracy of the former is also better than the latter, as shown in Table 5.

The initialization coordinates are mainly obtained by QR codes on the ground, and the initial position accuracy by using a visual location in the actual system is within 0.5 m; thus, $\Delta x_{u}$ is 0.5 m and $\Delta y_{u}$ is 0.5 m. The average velocity measurement errors caused by deviations of the receiver coordinates are about 10 mm/s for the two methods, as shown in Table 6. Therefore, the contributions of the two methods to the positioning accuracy are the same in actual use.

### 5.2. Deviation of the Receiver Antenna Coordinate

According to the geometric relationship between the transmitting antennas and receiver, we assume that the error of the receiver antenna coordinate is $\left[\begin{array}{ccc} \Delta x^{n} & \Delta y^{n} & \Delta z^{n} \end{array}\right]$, where *n* = 1,2,…,*n*. The Doppler observation equations (Equation (6)) can be written as

(24) $$\left[\begin{array}{ccc} e_{x}^{1} & e_{y}^{1} & e_{z}^{1}\\ e_{x}^{2} & e_{y}^{2} & e_{z}^{2}\\ \ldots & \ldots & \ldots \\ e_{x}^{n} & e_{y}^{n} & e_{z}^{n} \end{array}\right]\cdot \left[\begin{array}{c} {\dot{x}}_{u}\\ {\dot{y}}_{u}\\ {\dot{z}}_{u} \end{array}\right]+\underset{D}{\underset{︸}{\left[\begin{array}{ccc} \frac{\Delta x^{1}}{R_{u}^{1}} & \frac{\Delta y^{1}}{R_{u}^{1}} & \frac{\Delta z^{1}}{R_{u}^{1}}\\ \frac{\Delta x^{2}}{R_{u}^{2}} & \frac{\Delta y^{2}}{R_{u}^{2}} & \frac{\Delta z^{2}}{R_{u}^{2}}\\ \ldots & \ldots & \ldots \\ \frac{\Delta x^{n}}{R_{u}^{n}} & \frac{\Delta y^{n}}{R_{u}^{n}} & \frac{\Delta y^{n}}{R_{u}^{n}} \end{array}\right]\cdot \left[\begin{array}{c} {\dot{x}}_{u}\\ {\dot{y}}_{u}\\ {\dot{z}}_{u} \end{array}\right]}}=\left[\begin{array}{c} \frac{c}{f}\cdot D_{u}^{1}-(c\cdot d{\dot{t}}_{u}-c\cdot d{\dot{t}}^{s})\\ \frac{c}{f}\cdot D_{u}^{2}-(c\cdot d{\dot{t}}_{u}-c\cdot d{\dot{t}}^{s})\\ \ldots \\ \frac{c}{f}\cdot D_{u}^{n}-(c\cdot d{\dot{t}}_{u}-c\cdot d{\dot{t}}^{s}) \end{array}\right]$$

The differential positioning equation (Equation (9)) is denoted by

(25) $$\left[\begin{array}{ccc} e_{x}^{2}-e_{x}^{1} & e_{y}^{2}-e_{y}^{1} & e_{z}^{2}-e_{z}^{1}\\ e_{x}^{3}-e_{x}^{1} & e_{y}^{3}-e_{y}^{1} & e_{z}^{3}-e_{z}^{1}\\ \ldots & \ldots & \ldots \\ e_{x}^{n}-e_{x}^{1} & e_{y}^{n}-e_{y}^{1} & e_{z}^{n}-e_{z}^{1} \end{array}\right]\times \left[\begin{array}{c} {\dot{x}}_{u}\\ {\dot{y}}_{u}\\ {\dot{z}}_{u} \end{array}\right]+\underset{E}{\underset{︸}{\left[\begin{array}{ccc} \frac{\Delta x^{1}R_{u}^{2}-\Delta x^{2}R_{u}^{1}}{R_{u}^{1}R_{u}^{2}} & \frac{\Delta y^{1}R_{u}^{2}-\Delta y^{2}R_{u}^{1}}{R_{u}^{1}R_{u}^{2}} & \frac{\Delta z^{1}R_{u}^{2}-\Delta z^{2}R_{u}^{1}}{R_{u}^{1}R_{u}^{2}}\\ \frac{\Delta x^{1}R_{u}^{3}-\Delta x^{3}R_{u}^{1}}{R_{u}^{1}R_{u}^{3}} & \frac{\Delta y^{1}R_{u}^{3}-\Delta y^{3}R_{u}^{1}}{R_{u}^{1}R_{u}^{3}} & \frac{\Delta z^{1}R_{u}^{3}-\Delta z^{3}R_{u}^{1}}{R_{u}^{1}R_{u}^{3}}\\ \ldots & \ldots & \ldots \\ \frac{\Delta x^{1}R_{u}^{n}-\Delta x^{n}R_{u}^{1}}{R_{u}^{1}R_{u}^{n}} & \frac{\Delta y^{1}R_{u}^{n}-\Delta y^{n}R_{u}^{1}}{R_{u}^{1}R_{u}^{n}} & \frac{\Delta z^{1}R_{u}^{n}-\Delta z^{n}R_{u}^{1}}{R_{u}^{1}R_{u}^{n}} \end{array}\right]\cdot \left[\begin{array}{c} {\dot{x}}_{u}\\ {\dot{y}}_{u}\\ {\dot{z}}_{u} \end{array}\right]}}=\left[\begin{array}{c} \frac{c}{f}\cdot \Delta D_{u}^{2,1}\\ \frac{c}{f}\cdot \Delta D_{u}^{3,1}\\ \ldots \\ \frac{c}{f}\cdot \Delta D_{u}^{n,1} \end{array}\right]$$

**Figure 10:**
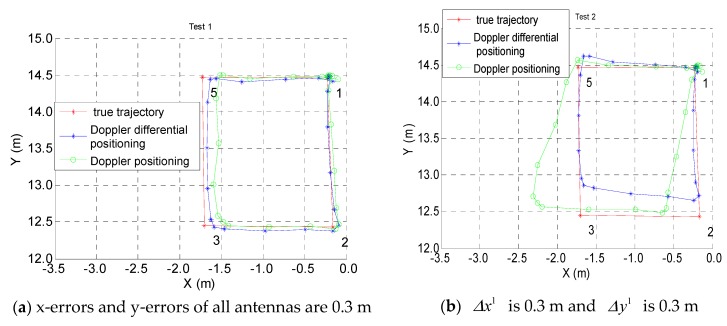

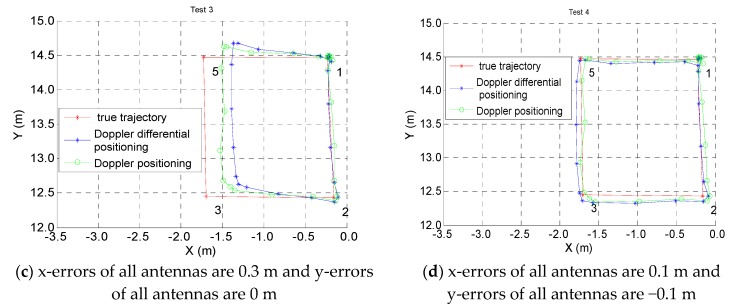
Positioning results with errors of the transmitting antenna coordinates.

The residual equations D and E are treated nonlinearly by the least squares updated solution (11), and it is very difficult to analyze the error by a mathematical method. The x-error or y-error, which ranges from 0.1 m to 0.3 m, is manually added to the coordinates of all transmitting antennas (the transmitting antennas in Table 1), and the data set for a kinematic test on a square trajectory in Figure 8 has been used. It can be found from Figure 10 that the deviation of the transmitting antenna will affect the positioning accuracy of the two methods, and if the differential positioning method cannot mitigate the antenna position error, the positioning accuracy of the differenced method is not necessarily better than that of the undifferenced method.

### 5.3. Horizontal Dilution of Precision (HDOP)

**Figure 11:**
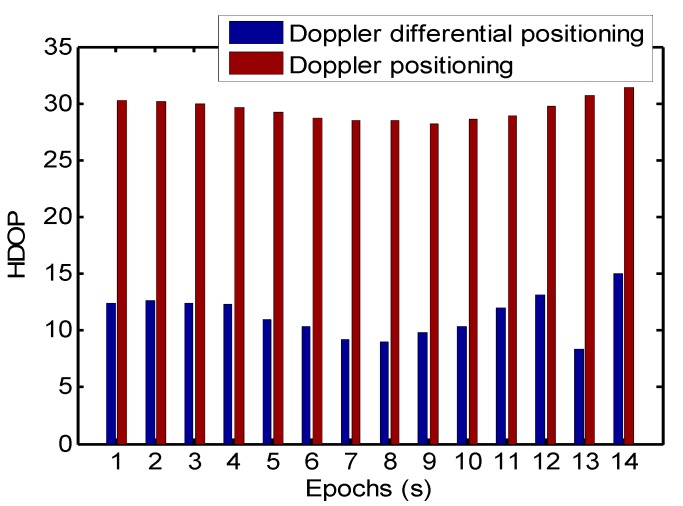
Horizontal dilution of precision (HDOP) on a straight-line trajectory.

Because of the narrow space, the aesthetic requirement of buildings, and constraints on equipment structure, it is difficult to get a good distribution for indoor array pseudolite; thus, a bad HDOP is mainly due to the distribution of pseudolite. The coordinates of the transmitting antennas are shown in Table 1, which are approximately distributed in a circular shape with the diameter of 3 m and are the same height from the ground. Figure 11 shows the horizontal dilution of precision on a straight-line trajectory in the kinematic test: the average HDOP of the Doppler differential positioning algorithm is 11.2, and the average HDOP of the Doppler positioning algorithm is 29.46.

**Figure 12:**
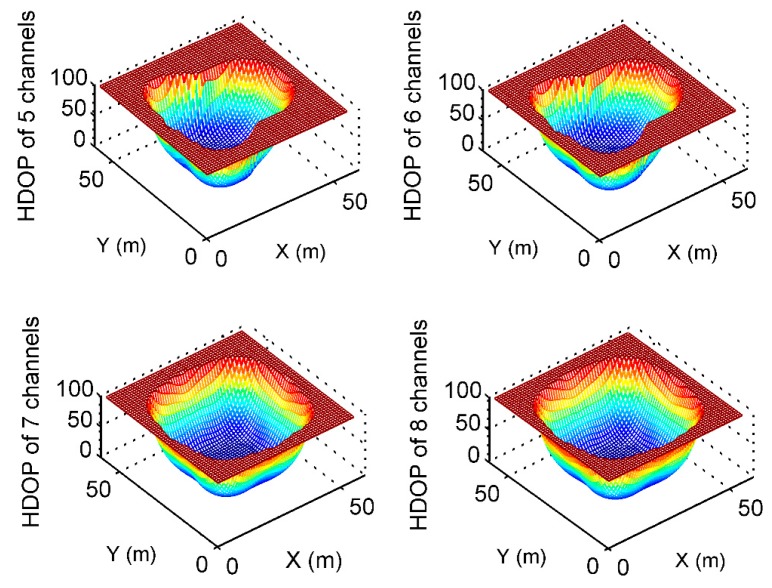
HDOP using Doppler positioning algorithm from 5 to 8 channels.

**Figure 13:**
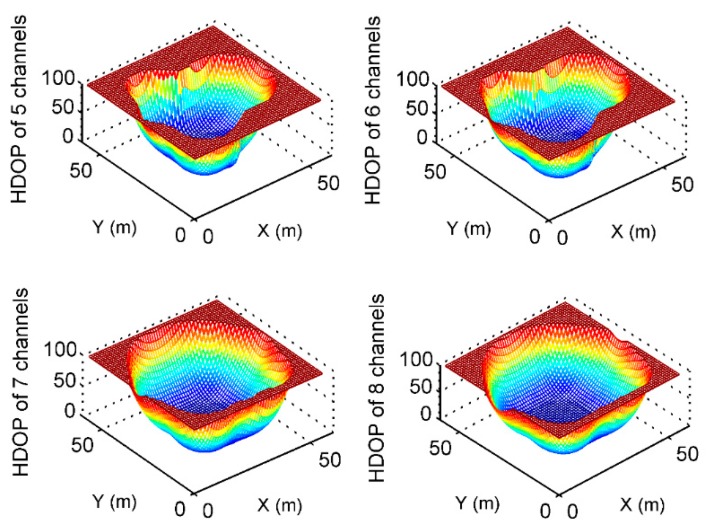
HDOP using Doppler differential positioning algorithm from channel 5 to channel 8.

According to the coordinates in Table 1, the HDOP values of the two positioning algorithms mentioned above are compared by a simulation; the area of the simulation analysis is 60 m × 60 m, and the interval is 1 m × 1 m. The coverage is analyzed under different numbers of transmission channels, as shown in Figure 12 and Figure 13. By counting the number of grids with an HDOP less than 100 in Table 7 from 5 to 8 transmitting channels of pseudolite, it can be found that the coverage of the Doppler differential positioning algorithm is better than that of the Doppler positioning algorithm, increasing from 15% to 20%.

**Figure 14:**
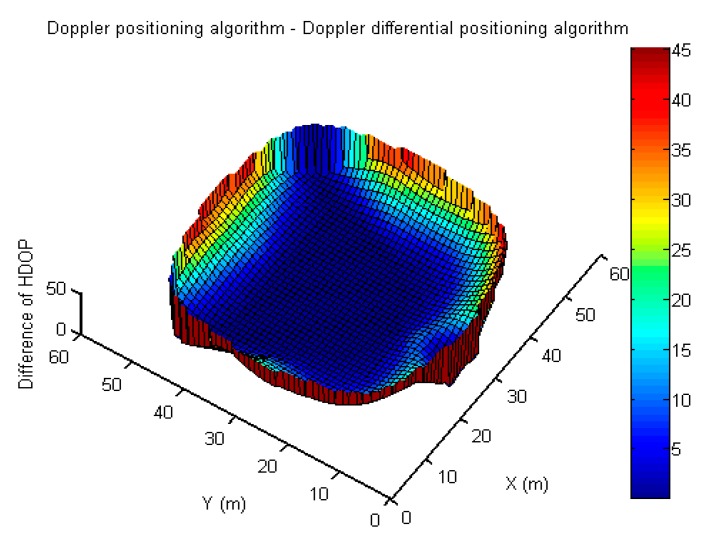
Difference of HDOP between the Doppler positioning algorithm and Doppler differential positioning algorithm.

**Figure 15:**
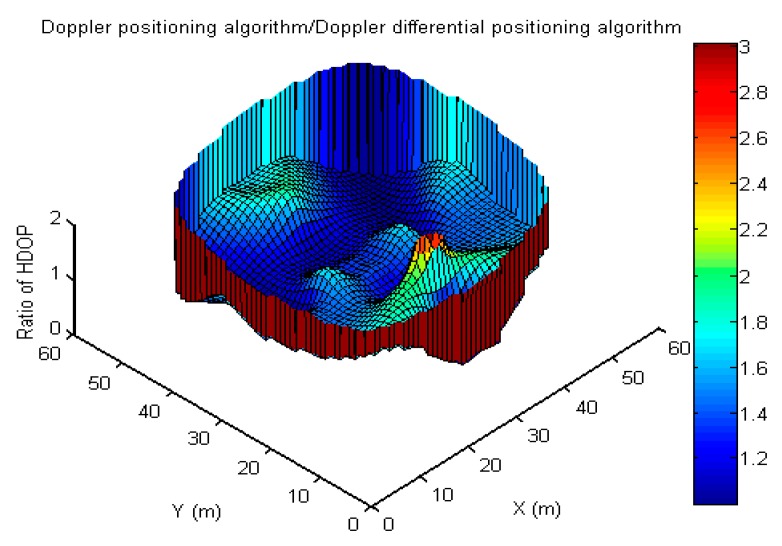
Ratio of HDOP between the Doppler positioning algorithm and Doppler differential positioning algorithm.

Because the size of the measurement noise also varies between the two methods, and the time dilution of precision (TDOP)-term in the differential method disappeared and was included in other DOPs, the performance of the two methods can hardly be analyzed with a mathematical discussion. In addition, because the errors of the antenna coordinates and time synchronization are reduced in the differential method but the errors of uncorrelated measurement are also increased, then, assuming that the measurement errors of the two methods are the same, the difference and ratio of the HDOP between the Doppler positioning algorithm and the Doppler differential positioning algorithm are used for qualitative analysis, as shown in Figure 14 and Figure 15. By counting the number of grids for the difference of HDOP (D_HDOP) in Table 8, and for the difference of HDOP (R_HDOP) in Table 9, it can be found that the HDOP of the Doppler positioning algorithm is greater than that of Doppler differential positioning algorithm; there are about 716 grids with D_HDOP greater than 10 and less than 50, accounting for about 33% of the total grids. There are about 852 grids with R_HDOP greater than 1.5, accounting for about 42% of the total grids. On the other hand, if the error term using differenced measurement may be 1.4 times of undifferenced measurements [24], the differenced method may improve the positioning accuracy in nearly 42% of the test area; however, the positioning accuracy of the differenced method may unfortunately not be better than that of the undifferenced method in nearly 58% of the test area.

### 5.4. Cycle-Slip Detection

The carrier phase jump will affect the accuracy of Doppler measurements, which will then affect the accuracy of indoor positioning. The causes of cycle-slips for carrier phase observations in the indoor environment are listed as below: (1) cycle-slips are caused by obstructions of the pseudolite signal due to the presence of buildings and pedestrians; and (2) cycle-slips have a low carrier-to-noise-power-density ratio (C/N0) due to multipath, near-far effect [25,26,27]. The carrier phase and Doppler measurement characteristics of indoor pseudolite (no ionospheric error, tropospheric error and time synchronization error) are different from GNSS. The Doppler-aided cycle-slip detection method (DACS) and the carrier phase double difference cycle-slip detection method (CPDD) are used.

Doppler shifts can be used to detect cycle-slips in carrier phase observations between neighboring epochs. The carrier phase observation at one epoch is predicted based on the Doppler shift and the carrier phase observation from the previous epoch.

(26) $${\bar{\phi }}_{u}^{i}(t+\Delta t)=\phi_{u}^{i}(t)+D_{u}^{i}(t)\times \Delta t$$

where ${\bar{\phi }}_{u}^{i}(t+\Delta t)$ is the predicted carrier phase observation from channel i to receiver *u* at epoch t+Δt, $\phi_{u}^{i}(t)$ is the carrier phase observation at epoch (t), $D_{u}^{i}(t)$ is Doppler observation at epoch t, and Δt is the time span between epoch (t+Δt) and epoch (t).

Doppler-aided cycle-slip detection can be expressed as

(27) $$\phi_{u}^{i}(t+\Delta t)-{\bar{\phi }}_{u}^{i}(t+\Delta t)<T$$

where $\phi_{u}^{i}(t+\Delta t)$ is the carrier phase observation at epoch (t+Δt), T is a cycle-slip detection threshold, and $\phi_{u}^{i}(t+\Delta t)-{\bar{\phi }}_{u}^{i}(t+\Delta t)$ is the deviations between the carrier phase observations from the receiver and those predicted from Doppler observations.

**Figure 16:**
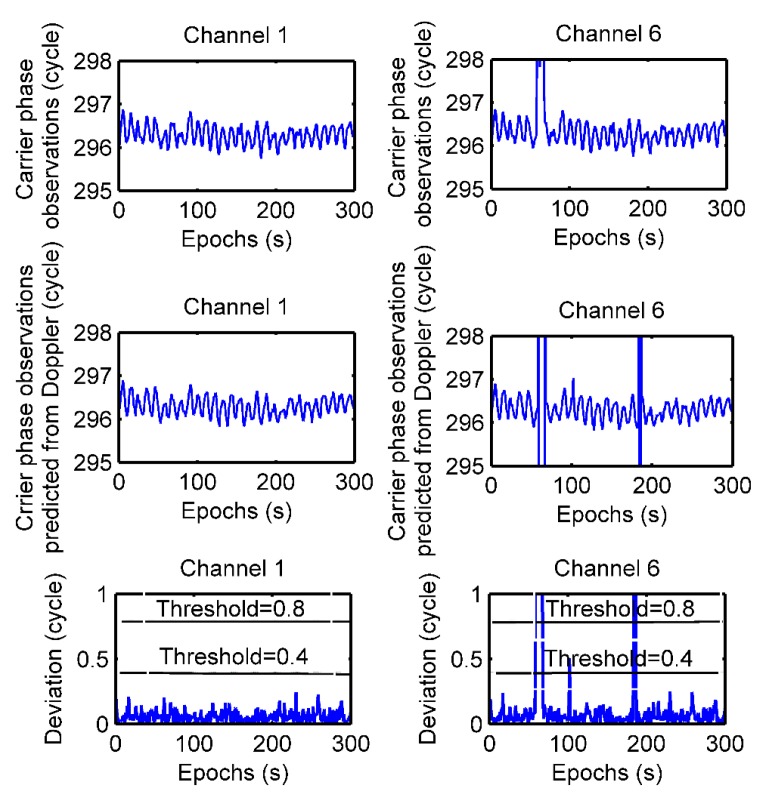
Static test of cycle-slip detection by the Doppler-aided cycle-slip detection method.

In the static test at test point 3, the raw data of channel 1 and channel 6 are analyzed regarding the performance of the cycle-slip detection approach. As shown in the top panel of Figure 16, there are no cycle-slips in channel 1, and there are some cycle-slips on channel 6. The deviations between carrier phase observations measured by a receiver and those predicted from Doppler observations are shown in the bottom panel of Figure 16; cycle-slips of one cycle or greater from epoch 59 to epoch 67 can be directly detected by the Doppler-aided cycle-slip detection method when the threshold is 0.8; the cycle-slips of a half-wavelength in epoch 102 can also be detected when the threshold is 0.4. 

**Figure 17:**
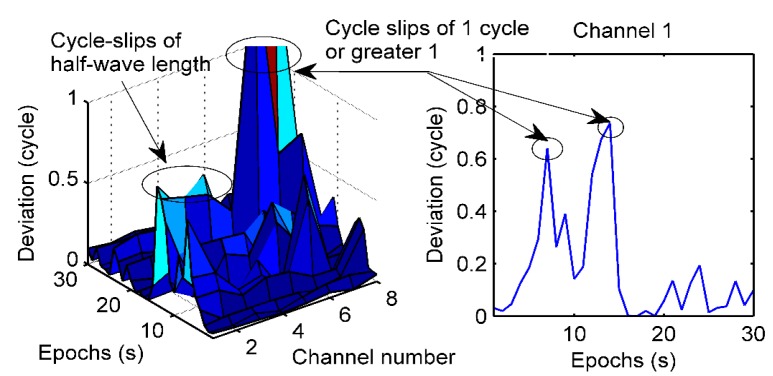
Kinematic test of cycle-slip detection by the Doppler-aided cycle-slip detection method.

The three-dimensional data of the Doppler-aided cycle-slip detection method for the kinematic test are shown in the left panel of Figure 17; the X-axis is the channel number of the pseudolite, the Y-axis shows epochs from 1 to 30, and the Z-axis shows the deviations between the carrier phase measured by a and predicted from the Doppler observations. In the kinematic test from epoch 1 to epoch 14, some cycle slips of one cycle or greater are at epoch 13 of channel 5 and epoch 14 of channel 6. Meanwhile, there are some deviations greater than a half-wavelength at epoch 7 and epoch 13, but these are mainly caused by the receiver’s speed, and not half-wavelength cycle-slips. In the static test from epoch 15 to epoch 30 in the right panel of Figure 17, all deviations are less than 0.4, and there is no cycle-slip. Therefore, it can be seen that the Doppler-aided cycle-slip detection method is suitable for detecting cycle-slips of one cycle or greater.

To detect a half-wavelength cycle-slip in kinematic positioning, the carrier phase double-difference cycle-slip detection method (CPDD) is proposed. The carrier phase difference equation between neighboring epochs can be expressed as

(28) $$\Delta \phi_{u}^{i}(t+\Delta t)=\phi_{u}^{i}(t+\Delta t)-\phi_{u}^{i}(t)$$

where $\Delta \phi_{u}^{i}(t+\Delta t)$ is the carrier phase difference equation between epoch (t) and epoch (t+Δt).

Then, the channel-difference of $\Delta \phi_{u}^{i}(t+\Delta t)$ between channel i and j can be expressed as

(29) $$\Delta \Delta \phi_{u}^{ij}(t+\Delta t)=\Delta \phi_{u}^{i}(t+\Delta t)-\Delta \phi_{u}^{j}(t+\Delta t)<T_{\Delta \Delta \phi }$$

where $T_{\Delta \Delta \phi }$ is the cycle slip detection threshold, and $\Delta \Delta \phi_{u}^{ij}(t+\Delta t)$ is the carrier phase double-difference observations, which can be used to detect cycle-slips of a half-wavelength.

**Figure 18:**
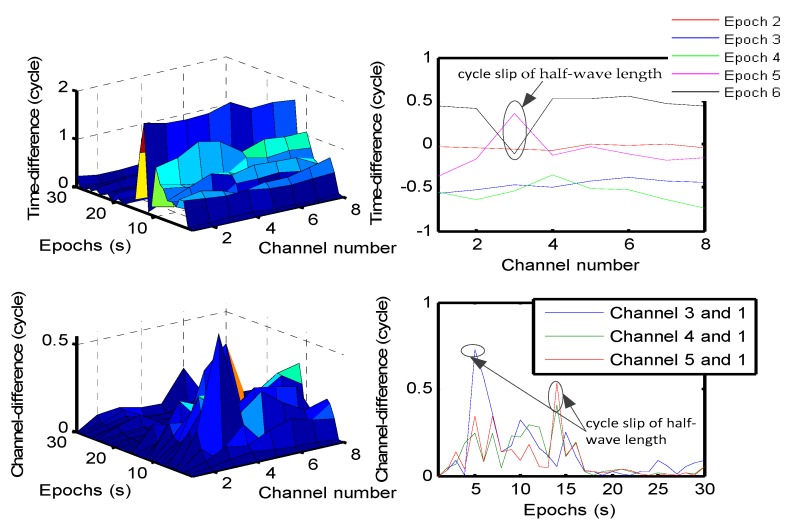
Kinematic experiment of cycle-slip detection by the carrier phase double-difference cycle-slip detection method.

In the kinematic test, one cycle-slip of a half-wavelength is manually inserted into the carrier phase measurements of channel 3 at epoch 5. Figure 18 shows the kinematic test of cycle-slip detection by the time-difference and channel-difference cycle-slip detection method, and the carrier phase differences between neighboring epochs are shown in the top panel of Figure 18. If there is no cycle-slip, the time difference of the carrier phase from channel 1 to channel 8 is nearly the same; for example, epoch 2, 3 and 4. If there are some cycle-slips, the time difference of the carrier phase for channel 3 at epoch 5 and epoch 6 may have a big “jump”. After the channel-difference, some half-wavelength cycle-slips can be determined at epoch 5 and 6; the results are shown in the bottom panel of Figure 18. This shows that the carrier phase double-difference cycle-slip detection method (CPDD) of the epoch which has an occurrence of a half-wavelength cycle-slip is much better than the others.

**Figure 19:**
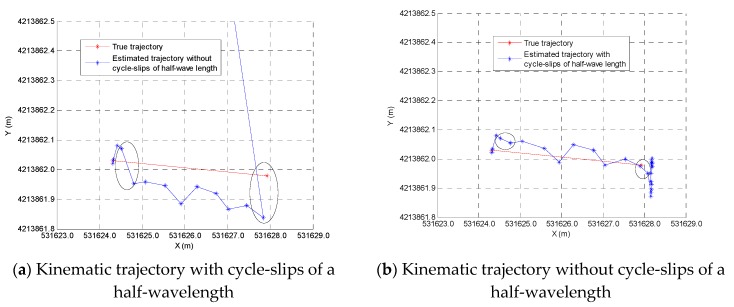
Kinematic trajectory with and without cycle-slips of a half-wavelength.

Figure 19 is the kinematic trajectory with and without cycle-slips of a half-wavelength. When a half-wavelength cycle-slip is manually inserted into the carrier phase measurements of channel 3 at epoch 5, some positioning errors begin to appear at epoch 4 in the absence of the detection of a half-wavelength cycle-slip; this shows that a half-wavelength cycle-slip can cause positioning errors of tens of millimeters (blue line in the left panel of Figure 19), which is the main positioning error of the BDS/GPS indoor array pseudolite system. The carrier phase double-difference cycle-slip detection method can detect cycle-slips of a half-wavelength in real time (blue line in the right panel of Figure 19). Therefore, the quality of Doppler measurements can be judged by the quality of carrier phase measurements, and cycle-slip detection methods are very important for the Doppler differential positioning algorithm of pseudolite.

## 6. Conclusions

In this work, a high-accuracy indoor positioning system based on BDS/GPS indoor array pseudolite is introduced, and a multi-channel transmitter with the same clock source is used to reduce the difficulty and complexity of time synchronization. Each channel transmits a navigation signal with different spread spectrum codes from 173 to 182 and a navigation message by modulating them at 1575.42 MHz and 1561.098 MHz. The Doppler differential positioning algorithm is proposed to improve indoor positioning accuracy and coverage, which uses the Doppler difference equation and Known Point Initialization (KPI) to determinate the velocity and position of the receiver. To enhance the indoor positioning accuracy, a Doppler-aided cycle-lip detection method (DACS) and carrier phase double-difference cycle-slip detection method (CPDD) are also used. According to the static and kinematic test, the results show that (1) the average positioning error of the Doppler differential positioning algorithm is 7.86 mm in the kinematic test and 2.9 mm in the static test, while the BDS/GPS indoor array pseudolite system has the potential to allow indoor positioning with sub-centimeter precision. (2) A half-wavelength cycle-slip can cause positioning errors of tens of millimeters; the Doppler-aided cycle-slip detection method can detect cycle slips of one cycle or greater, and the carrier phase double-difference cycle-slip detection method can detect cycle-slips of a half-wavelength. In future, we will focus on the study of the indoor array pseudolite/ wireless fidelity with round-trip time (WIFI-RTT) integrated location system, while pseudo-range observations of WIFI-RTT and carrier phase observations of pseudolite will be used to calculate the coordinates of initialization.

## Figures and Tables

**Table 1:** Coordinates of eight transmitting antennas.

	X	Y	Z
Transmitting Antenna 1	531627.37	4213862.35	88
Transmitting Antenna 2	531626.87	4213863.51	87.91
Transmitting Antenna 3	531625.88	4213863.89	87.83
Transmitting Antenna 4	531624.93	4213863.48	87.86
Transmitting Antenna 5	531624.41	4213862.64	88.02
Transmitting Antenna 6	531624.76	4213861.33	87.99
Transmitting Antenna 7	531625.90	4213860.82	88.13
Transmitting Antenna 8	531626.87	4213861.14	88.04

**Table 2:** Average value (AVG) and standard deviation (STD) of Doppler differential measurements in the static test.

	Doppler Differential Measurements (m/s)				
	$\Delta D_{u}^{2,1}$	$\Delta D_{u}^{3,1}$	$\Delta D_{u}^{4,1}$	$\Delta D_{u}^{5,1}$	$\Delta D_{u}^{6,1}$
AVG	−2.4 × 10^−^^6^	4.9 × 10^−6^	−2.2 × 10^−5^	1.2 × 10^−6^	3.4 × 10^−5^
STD	3.5 × 10^−^^3^	5.0 × 10^−3^	3.7 × 10^−3^	2.4 × 10^−3^	5.0 × 10^−3^

**Table 3:** Kinematic positioning error on a straight-line trajectory.

Epoch	Kinematic Positioning Error (mm)												
	1	2	3	4	5	6	7	8	9	10	11	12	13
Doppler Differential Positioning	0	7	26	41	28	43	1	11	2	15	0	−34	−48
Doppler Positioning	0	8	27	43	28	43	1	11	54	15	31	2	11

**Table 4:** Kinematic positioning error on a square trajectory.

**Epoch**	**Kinematic Positioning Error (mm)**											
	**1**	**2**	**3**	**4**	**5**	**6**	**7**	**8**	**9**	**10**	**11**	**12**
Doppler Differential Positioning	0	10	20	40	3	0	0	0	30	80	30	40
Doppler Positioning	0	10	20	40	30	0	20	30	40	80	10	20
**Epoch**	**13**	**14**	**15**	**16**	**17**	**18**	**19**	**20**	**21**	**22**	**23**	**24**
Doppler Differential Positioning	70	40	20	7	20	10	10	20	20	10	10	10
Doppler Positioning	40	30	30	120	70	130	10	50	50	0	30	20

**Table 5:** Velocity measurement error caused by deviations of the receiver coordinates.

Epoch $(\Delta x_{u}$$\;=\;4.0\;m,\;\Delta y_{u}=\;4.0\;m)$	Velocity Measurement Error (mm/s)					
	2	4	6	8	10	12
Doppler Positioning	73	115	890	936	116	290
Doppler Differential Positioning	68	99	862	872	97	270
$\Delta V_{Doppler\;positioning}-\Delta V_{Doppler\;differential\;positioning}$	5	16	28	64	19	20
**Epoch**	**14**	**16**	**18**	**20**	**22**	**24**
Doppler Positioning	78	21	702	1719	3438	13,707,200
Doppler Differential Positioning	34	9	545	1415	3250	7839
$\Delta V_{Doppler\;positioning}-\Delta V_{Doppler\;differential\;positioning}$	44	12	157	304	188	13,699,361

**Table 6:** Velocity measurement error caused by deviations of the receiver coordinates.

**Epoch** ($\Delta x_{u}$ = 0.5 m, $\Delta y_{u}$= 0.5 m)	**Velocity Measurement Error (mm/s)**					
	**2**	**4**	**6**	**8**	**10**	**12**
Doppler Positioning	1	2	9	8	5	16
Doppler Differential Positioning	1	2	7	6	5	12
$\Delta V_{Doppler\;positioning}-\Delta V_{Doppler\;differential\;positioning}$	0	0	2	2	0	3
**Epoch**	**14**	**16**	**18**	**20**	**22**	**24**
Doppler Positioning	19	9	14	8	29	**10**
Doppler Differential Positioning	19	8	12	7	24	8
$\Delta V_{Doppler\;positioning}-\Delta V_{Doppler\;differential\;positioning}$	0	1	2	1	3	2

**Table 7:** Number of grids for HDOP with the different number of transmitting Channels.

HDOP < 100	Number of Channels for BDS/GPS Indoor Array Pseudolite			
	5	6	7	8
Doppler Differential Positioning Algorithm	1708	1819	2326	2447
Doppler Positioning Algorithm	1467	1536	1969	2035
Proportion	1.164	1.184	1.181	1.20

**Table 8:** Number of grids for the difference of HDOP (D_HDOP).

	0 < D_HDOP ≤ 1	1 < D_HDOP < 10	10 ≤ D_HDOP < 50
Number of grids	676	643	716
Proportion	0.33	0.32	0.35

**Table 9:** Number of grids for the ratio of HDOP (R_HDOP).

	R_HDOP≤ 1	1 < R_HDOP < 1.5	1.5 ≤ R_HDOP < 2	2 ≤ R_HDOP
Number of Grids	0	1183	810	42
Proportion	0	0.58	0.40	0.02
